# Neuralgia of the Inferior Maxillary Nerve, Cured by Trephining the Jaw, and Excision of the Nerve

**Published:** 1858-08

**Authors:** Frank H. Hamilton

**Affiliations:** Buffalo


ART. IV.—*Neuralgia of the Inferior Maxillary Nerve, cured by Trephining the Jaw, and Excision of the Nerve.* By FRANK H. HAMILTON, M. D., Buffalo.

Finla Goff, of Addison, Steuben Co., N. Y., aged about thirty years, had suffered from neuralgia in the right side of the face and jaw, for a period of seven or eight years. The pain originated, apparently, in the last molar of that side; the tooth had long since been removed, but no relief to the neuralgia. Only once or twice during this period had he experienced a cessation of pain. During the first few months which he spent in California, he was without pain, but it returned, after a severe exposure, and has never been well since. The paroxysms of pain were brief, but terrible; occurring every few minutes, both day and night.

April 30th, 1858. I operated, assisted by Dr. Boardman and others. Having exposed the bone opposite the angle of the inferior maxilla, I applied a medium sized trephine, and perforated completely the jaw, removing with the section of bone about three-quarters of an inch of the inferior maxillary nerve. Finding that the pain still continued to occur in the skin of the chin, I severed the mucous membrane and the muscles freely.

In removing the trephine, the narrow vinculum of the jaw which remained, was broken, and this I was obliged to close, by perforating the two ends and securing them together with silver wire. No splints were applied.

June 14, 1858. Six weeks after the operation was made, I received the following letter, which I enclose to you because it is an account of his original sufferings and of the amount of relief which he has experienced, given by one who has been a witness of the whole:

CAMERON MILLS, June 14th, 1858.

F. H. HAMILTON, M. D.,

Dear Sir: By request of my brother-in-law, Mr. Finla Goff, I address you at this time to inform you of his present state of health. I am happy to be able to make a good report of his case. Thus far he has been *perfectly free* from *pain*, except a few twinges about the point of the chin, when he first returned. The jaw has united quite firmly; the wire broke off close to the jaw some three weeks since. The wound has closed up within a few days, and Mr. Goff started for Addison this morning to engage in his regular employment, and I can assure you he is a happy man—says he would be willing to endure the same operation, if necessary, every six months, to be as free from pain as he is at present. He has gained very materially in flesh and feels very cheerful; says he feels quite confident the cure will be permanent.

It must afford you much satisfaction to feel that you have been instrumental in thus relieving one of your fellow men from the most exquisite suffering to which human flesh is subject. I have seen his sufferings until my heart truly bled for him, and I greatly rejoice to see him so perfectly relieved.

Very respectfully yours,

S. MITCHELL.

